# The Glycoproteins of All Filovirus Species Use the Same Host Factors for Entry into Bat and Human Cells but Entry Efficiency Is Species Dependent

**DOI:** 10.1371/journal.pone.0149651

**Published:** 2016-02-22

**Authors:** Markus Hoffmann, Mariana González Hernández, Elisabeth Berger, Andrea Marzi, Stefan Pöhlmann

**Affiliations:** 1 Infection Biology Unit, German Primate Center, Göttingen, Germany; 2 Laboratory of Virology, Division of Intramural Research, National Institute of Allergy and Infectious Diseases, National Institutes of Health, Hamilton, Montana, United States of America; Thomas Jefferson University, UNITED STATES

## Abstract

Ebola and marburgviruses, members of the family *Filoviridae*, can cause severe hemorrhagic fever in humans. The ongoing Ebola virus (EBOV) disease epidemic in Western Africa claimed more than 11,300 lives and was associated with secondary cases outside Africa, demonstrating that filoviruses pose a global health threat. Bats constitute an important natural reservoir of filoviruses, including viruses of the recently identified *Cuevavirus* genus within the *Filoviridae* family. However, the interactions of filoviruses with bat cells are incompletely understood. Here, we investigated whether filoviruses employ different strategies to enter human and bat cells. For this, we examined host cell entry driven by glycoproteins (GP) from all filovirus species into cell lines of human and fruit bat origin. We show that all GPs were able to mediate entry into human and most fruit bat cell lines with roughly comparable efficiency. In contrast, the efficiency of entry into the cell line EidNi/41 derived from a straw-colored fruit bat varied markedly between the GPs of different filovirus species. Furthermore, inhibition studies demonstrated that filoviruses employ the same host cell factors for entry into human, non-human primate and fruit bat cell lines, including cysteine proteases, two pore channels and NPC1 (Niemann-Pick C1 molecule). Finally, processing of GP by furin and the presence of the mucin-like domain in GP were dispensable for entry into both human and bat cell lines. Collectively, these results show that filoviruses rely on the same host cell factors for entry into human and fruit bat cells, although the efficiency of the usage of these factors might differ between filovirus species.

## Introduction

Filovirus infection can cause a life threatening hemorrhagic fever (e.g. Ebola virus disease, EVD) in non-human primates (NHP) and humans, with case-fatality rates of up to 90%. Before 2013, filovirus outbreaks in human populations were restricted to remote areas in Central Africa and were associated with less than 500 cases. The Ebola virus (EBOV) outbreak in Guinea in 2013 resulted for the first time in viral spread from rural to densely populated areas and had severe consequences: The Ebola virus disease (EVD) epidemic affected major cities in Guinea, Liberia and Sierra Leone and caused 11,312 deaths (as of 11 October 2015). Moreover, secondary infections occurred in countries not hit by the epidemic, including the USA and Spain. Thus, filoviruses constitute a global health threat.

The family of *Filoviridae* contains three genera, *Ebolavirus*, *Marburgvirus* and *Cuevavirus*. The only species within the Marburgvirus genus, *Marburg marburgvirus* (members: Marburg virus, MARV and Ravn virus, RAVV), and the following ebolaviruses are pathogenic to humans: Ebola virus (EBOV, species *Zaire ebolavirus*), Sudan virus (SUDV, species *Sudan ebolavirus*, Bundibugyo virus (BDBV, species *Bundibugyo ebolavirus*) and Taï Forest virus, (TAFV, species *Taï Forest ebolavirus*). Reston virus (RESTV), the only member of the fifth ebolavirus species, causes disease in NHP but has so far not been associated with disease in humans. Moreover, RESTV infection of pigs and bats has been documented [1, 2]. The genome of Lloviu virus (LLOV), the only virus within the species *Lloviu cuevavirus*, genus *Cuevavirus*, has been detected in dead bats (*Miniopteris schreibersii*) in Northern Spain [3], its potential to infect humans is unknown.

Bats, especially fruit bats (family *Pteropodidae*), are considered a natural reservoir for filoviruses from which these viruses might be transmitted to humans either directly or via intermediate hosts (e.g. antelopes or primates) [4–6]. The role of bats as a natural reservoir for filoviruses is supported by the (i) detection of filoviruses in multiple, geographically dispersed bat species (on serological and nucleic acid level), (ii) isolation of MARV-like viruses from apparently healthy Egyptian fruit bats (*Rousettus aegyptiacus*, so far EBOV-like viruses were not isolated from bats), and (iii) the observation that experimentally infected bats shed the virus but do not develop clinical symptoms [3, 7–16]. The association of filoviruses with bats does not come as a surprise, since also for other emerging zoonotic viruses multiple bat species have been proven or are believed to serve as a natural reservoir (e.g. reviewed in [17, 18]). However, the interactions of filoviruses with bat cells are incompletely understood. In particular, the processes underlying filovirus entry into bat cells are largely unknown.

**Fig 1:**
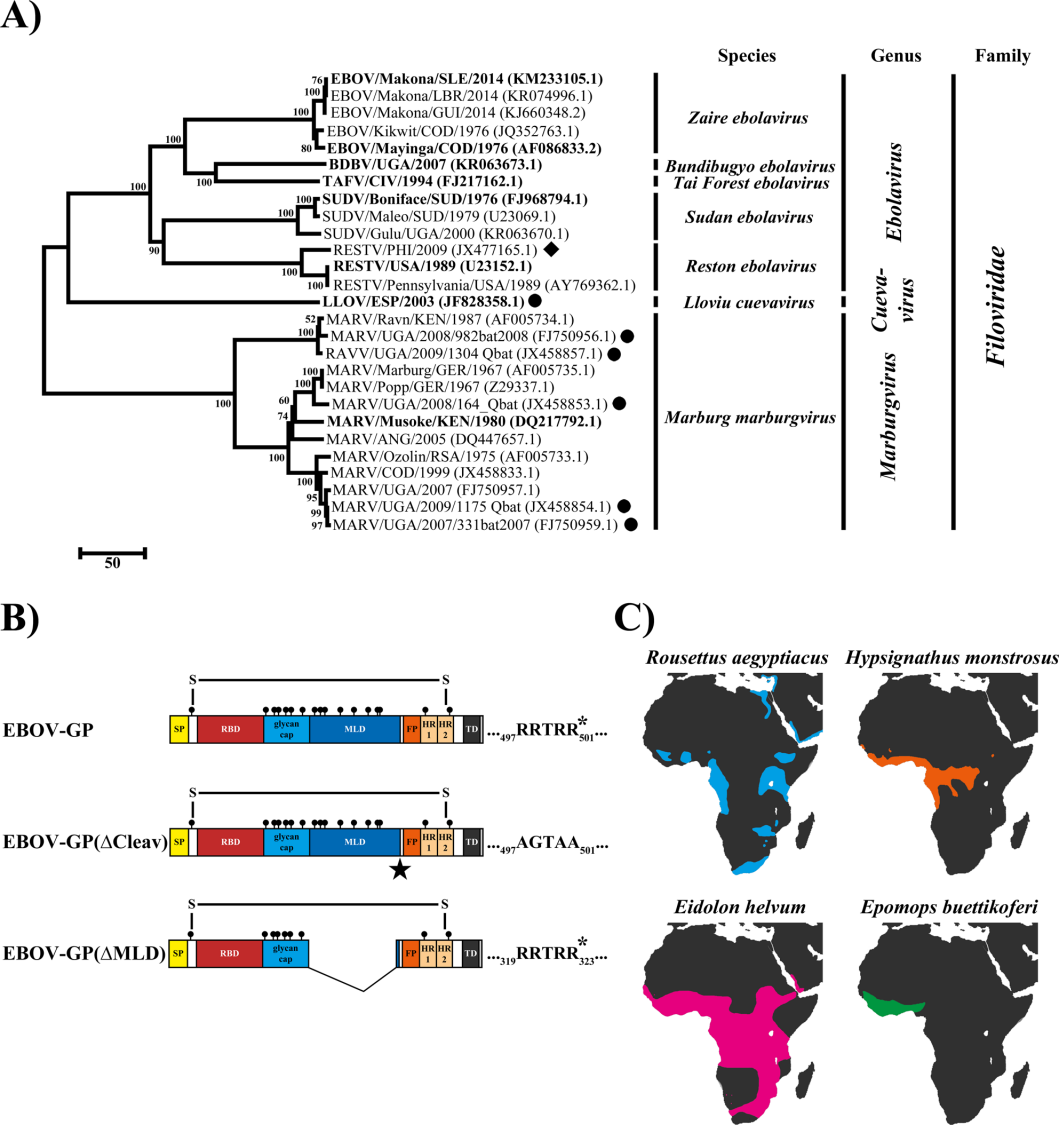
Filovirus glycoproteins and distribution of fruit bats believed to serve as natural reservoir. (A) Phylogenetic tree based on the amino acid (aa) sequences of the glycoproteins (GP) of different filoviruses was generated using MEGA 6 (version 6.06). Viruses from which GPs were examined in the present study are written in bold. Viruses were named as follows: Filovirus species (abbreviation)/Country where the sample specimen originates from (abbreviation)/year of sampling/isolate-specific name (if available). In addition to GPs from viruses that have caused infection in humans and non-human primates, GP sequences of one Reston virus from a pig (diamond), as well as four Marburg virus-related and two Ravn virus-related GP sequences from bats (circles) were included. Construction of the tree was performed by the neighbor joining method with 1,000 bootstrap replications, using the MEGA 6 software (version 6.06). Small numbers at the nodes and the scale bar indicate bootstrap values and the number of aa substitutions per site, respectively. GenBank accession numbers for all GPs are given in brackets after the virus name. (B) Schematic drawing of the wildtype (wt) Ebola virus (EBOV) GP and EBOV-GP mutants lacking the mucin-like domain and the furin cleavage site. (Abbreviations: SP = signal peptide; RBD = receptor binding domain; FP = internal fusion peptide; HR1/2 = heptad repeat 1/2; TD = transmembrane domain). (C) Maps of the distribution of the four different fruit bat species from which the cell lines used in this study originated: *Rousettus aegyptiacus* (RoNi/7), *Hypsignathus monstrosus* (HypNi/1.1), *Eidolon helvum* (EidNi/41), *and Epomops buettikoferi* (EpoNi/22.1). The data on the bat distribution have been obtained from www.iucnredlist.org [56].

The filovirus glycoprotein (GP) is the only viral protein embedded in the viral envelope and constitutes the sole determinant of host cell entry [19]. In addition, the GP is a virulence factor [20, 21]. The filovirus GP is synthesized as a precursor protein (GP_0_), which is extensively modified by N- and O-glycans and processed by subtilisin-like proprotein convertases (especially furin) during passage through the secretory pathway [22]. Cleavage occurs between the surface unit, GP1, which contains the receptor binding domain (RBD) and a mucin-like domain (MLD), and the transmembrane unit, GP2, which anchors the GP in the viral envelope and harbors the membrane fusion machinery (Fig 1B). Although the cleavage motifs for proprotein-convertases are found in all filovirus GPs (RESTV-GP contains a non-classical furin recognition motif [22]), processing of EBOV-GP by these enzymes is dispensable for robust viral spread in cell culture and in the host [23, 24].

Filovirus entry into human cells starts with the attachment of GP to molecules on the cell surface, such as lectins or the human T cell immunoglobulin mucin 1 (TIM-1) surface molecule [25–28]. In addition, signaling factors like members of the TAM (Tyro3/Axl/Mer) family of tyrosine kinase receptors and α5β1-Integrin have been shown to promote filovirus entry [29–31]. Upon uptake of EBOV particles into endosomes, the activity of two-pore channels (TPCs, endosomal calcium channels) [32] and GP processing by endosomal pH-dependent cysteine proteases cathepsin B/L are believed to be required for the entry cascade to progress [33–35], at least in several cell culture systems. Cleavage removes the MLD and allows binding of the RBD to NPC1 [36, 37]. Subsequently, an incompletely understood trigger is required to start the GP2-driven fusion of the viral and the endosomal membrane, which allows for the release of the viral genetic information in the host cell cytoplasm. Whether the same molecular mechanisms underlie filovirus entry into bat cells is largely unknown, although LLOV entry into human and NHP cells has been reported to depend on NPC1 and cathepsins [38].

We comparatively analyzed filovirus GP-driven entry into human, NHP and bat cell lines. The latter were generated from bat species that are believed to constitute the natural reservoir of filoviruses. Our results show that filoviruses use the same entry mechanism for infection of human, NHP and bat cells. However, the efficiency with which the glycoproteins of certain filovirus species interact with host cell factors during the entry process might differ.

## Materials and Methods

### Cell culture

The following cell lines were used as targets for transduction experiments and were maintained in Dulbecco's modified Eagle's medium (PAA Laboratories), supplemented with 10% fetal bovine serum (Biochrom) and antibiotics: HEK-293T (human embryonic kidney; ATCC CRL-3216), Vero (African green monkey, kidney; ATCC CCL-81) as well as chiropteran cell lines from four different species of fruit bats (Table 1), RoNi/7 (*Rousettus aegyptiacus*, kidney), HypNi/1.1 (*Hypsignathus monstrosus*, kidney) EidNi/41 (*Eidolon helvum*, kidney) and EpoNi/22.1 (*Epomops buettikoferi*, kidney). A baby hamster kidney cell line (BHK-21; DSMZ No. ACC-61) was solely used for the production of rhabdoviral pseudotypes and was maintained as described for the other cell lines. All cell lines were obtained from collaborators. The fruit bat cell lines were a kind gift of C. Drosten and M. A. Müller (University of Bonn Medical Centre, Bonn/Germany) and were previously described [39–43]. All cell lines were grown in a humidified atmosphere at 37°C and 5% CO_2_. For passaging and seeding, cells were detached by either resuspension in fresh culture medium (HEK-293T cells) or the use of trypsin/EDTA (PAA Laboratories; Vero, BHK-21, and bat cells).

**Table 1:** Information on the fruit bat cell lines used in this study.

Name	Species	Organ	Immortalization method
RoNi/7	Egyptian fruit bat (*Rousettus aegyptiacus*)	Kidney	Simian virus 40 large T antigen
HypNi/1.1	Hammer-headed fruit bat (*Hypsignathus monstrosus*)	Kidney	Simian virus 40 large T antigen
EidNi/41	Straw-colored fruit bat (*Eidolon helvum*)	Kidney	Simian virus 40 large T antigen
EpoNi/22.1	Buettikofer's epauletted fruit bat (*Epomops buettikoferi*)	Kidney	Simian virus 40 large T antigen

### Plasmids

The open reading frames (ORF) for 1976 Ebola virus (EBOV1976, strain Mayinga; GenBank accession number: AF086833.2), 2014 Ebola virus (EBOV2014, Makona variant; KM233105) Sudan virus (SUDV, strain Boniface; FJ968794.1), Bundibugyo virus (BDBV; KR063673.1), Taï Forest virus (TAFV; FJ217162.1) or Reston virus (RESTV; U23152.1) glycoprotein (GP) were amplified from existing expression plasmids [26, 27, 44–47] by polymerase chain reaction (PCR) and inserted into the pCAGGS vector [48] by conventional cloning strategies. Details on the cloning strategy (e.g. restriction sites and primer sequences) are available upon request. Expression plasmids (pCAGGS-based) for Marburg virus (MARV, strain Musoke; DQ217792.1) and Lloviu virus (LLOV; JF828358.1) were kindly provided by S. Becker (Philipps-University Marburg, Marburg/Germany) and A. Takada (Hokkaido University Research Center for Zoonosis Control, Sapporo/Japan), respectively [49, 50]. In addition, mutant EBOV-GP in which the furin cleavage motif has been destroyed (EBOV-GP(ΔCleav)) or most of the amino acid (aa) residues comprising the mucin-like domain (MLD, aa residues 309–486) have been deleted (EBOV-GP(ΔMLD)) were constructed by overlap extension PCR from the plasmid containing the wildtype (wt) EBOV-GP sequence. The expression plasmid for the glycoprotein (G) of vesicular stomatitis virus (VSV, Indiana strain, VSV-G; AJ318514.1) was generated by inserting the VSV-G ORF into the pCG1 expression vector and has been used in previous studies [39, 42, 51, 52].

### Construction of phylogenetic trees

Phylogenetic analysis was performed based on the aa sequences of the filovirus GPs that were subject of this study as well as reference sequences from the National Center for Biotechnology Information (NCBI) database, using the MEGA 6 (version 6.06) software [53]. All GP sequences were first aligned using the MUSCLE algorithm before a phylogenetic tree was constructed based on the neighbor-joining method with 1,000 bootstrap iterations. The GenBank accession numbers for all sequences are stated in Fig 1.

### Production of rhabdoviral pseudotypes

For the generation of rhabdoviral pseudotypes bearing heterologous viral glycoproteins we employed a replication-deficient VSV vector. Instead of the genetic information for VSV-G, the vector contains two separate open reading frames coding for enhanced green fluorescent protein (EGFP) and firefly luciferase (fLuc), VSV*ΔG-fLuc [39, 42, 52]. The vector was propagated in a previously described VSV-G-expressing, transgenic cell line [54]. A detailed protocol for the production of pseudotyped VSV (VSVpp) can be found elsewhere [39]. In brief, BHK-21 cells were transfected with expression plasmids for VSV-G (positive control), the respective filovirus glycoproteins or empty plasmid (pCAGGS; negative control) using Lipofectamine2000 (Thermo Fisher Scientific) according to manufacturer’s protocol. At 16 h post transfection, the cells were first inoculated with VSV*ΔG-Luc at a multiplicity of infection of 3, then incubated with a polyclonal anti-VSV serum to block the infectivity of residual input virus (both for 1 h at 37°C and 5% CO_2_). Finally, the cells received fresh culture medium and were further incubated for 16–20 h, before the VSVpp-containing supernatants were collected, centrifuged (to remove cell debris) and aliquoted. Aliquots were stored at 4°C for a maximum of 7 days.

### Treatment of cell lines with chemical substances and inhibitors

The impact of the following compounds on filovirus GP-driven entry was studied (for detailed information please refer to Table 2): Mannan, ammonium chloride, bafilomycin A1, E-64d, camostat mesylate (all Sigma-Aldrich), tetrandrine, MDL28170 (both Abcam) and U18666A (Merck Millipore). For convenient use, 100–1,000x stock solutions of mannan, ammonium chloride, camostat mesylate and U18666A were prepared in water, while bafilomycin A1, tetrandrine, E-64d and MDL28170 were dissolved in dimethyl sulfoxide (DMSO). Directly before use, all compounds were diluted in culture medium to yield their respective working concentrations. For inhibition experiments, target cells were incubated in the presence of either compound or diluent (vehicle control, VC; water or DMSO) for 3 h at 37°C and 5% CO_2_. Then, the culture medium was removed, the cells were washed with phosphate buffered saline (PBS; 137 mM NaCl, 2.7 mM KCl, 10 mM Na_2_HPO_4_, 1.8 mM KH_2_PO_4_) and subsequently inoculated with VSVpp for 1 h as described below. After inoculation, the cells were further incubated in the presence of the respective compound or VC for 16–18 h at 37°C and 5% CO_2_.

**Table 2:** Overview on the chemical substances/inhibitors used in this study and their mode of action.

Substance/Inhibitor	Mode of action	Concentration	Reference(s)
Mannan	Blocks access to cell surface mannose-binding lectins	25 μg/ml	[62]
Ammonium chloride	Interferes with endosomal acidification (acts as a weak base)	50 mM	[63]
Bafilomycin A1	Interferes with endosomal acidification (inhibitor of vacuolar ATPases)	50 nM	[64]
Tetrandrine	Blocks two-pore channels	2 μM	[32]
E-64d	Inhibitor of cathepsins B and L, and calpain	50 μM	[35]
MDL28170	Inhibitor of calpain and cathepsin B	50 μM	[65]
Camostat mesylate	Serine protease inhibitor	100 μM	[66–68]
U18666A	Induces cholesterol accumulation in endosomes	20 μM	[37, 69]

### Transduction of cell lines with rhabdoviral pseudotypes and quantification of fLuc activity

For the transduction of cell lines (with or without prior treatment), the cells were seeded in 96-well plates (Thermo Scientific). At 24 h post seeding, the cell culture medium was removed and the cells were washed with PBS. Then, either undiluted or infectivity-normalized VSVpp were added (in quadruplicates) and the cells were further incubated for 1 h at 37°C and 5% CO_2_. Afterwards, the inoculum was removed and the cells were again washed with PBS and incubated with fresh culture medium for 16–18 h at 37°C and 5% CO_2_. To produce infectivity-normalized pseudotypes, the transduction efficiency (fLuc activity) was quantified in HEK-293T cells and the VSVpp were diluted in cell culture medium to yield comparable luciferase activity.

For the quantification of the fLuc activity as an indicator of transduction efficiency, the cell culture supernatant was removed and the cells were washed with PBS. Next, 50 μl of 1x solution of 5x Luciferase Cell Culture Lysis Reagent (Promega) in PBS was added to each well and incubated for 30 min at room temperature, before the cell lysate was transferred to a white, opaque-walled 96well plate (Thermo Scientific). The measurement of the fLuc activity was carried out in a microplate reader, Plate CHAMELEON (Hidex), using the MicroWin 2000 software (version 4.44, Mikrotek Laborsysteme GmbH) and fLuc substrates from the Luciferase Assay System (Promega) or Beetle-Juice (PJK) kits. Transduction efficiency, represented by fLuc activity, is either given in counts per second (cps) or displayed as x-fold change normalized against a control. All fLuc-assays were performed with quadruplicate samples.

### Statistical analysis

In order to assess statistical significance, an unpaired (for single representative experiments) or paired (for graphs showing the mean of three independent experiments) student’s t-test was performed. For inhibitor studies, statistical significance was calculated for results differing at least 5-fold from control conditions, since smaller effects were considered to be within the variation inherent to the assay system.

## Results

### Glycoproteins of different filovirus species mediate entry into certain bat cell lines with differential efficiency

To address whether the molecular mechanism of how filoviruses enter human cells also applies to cells from bats, we first assessed the susceptibility of fruit bat cell lines to transduction mediated by filovirus GPs. For this, rhabdoviral pseudotypes were employed that were decorated with GPs representative of all filovirus species (Fig 1A). We included two representatives for the *Zaire ebolavirus* species, EBOV-GP obtained during the EVD outbreak of 1976 in the Democratic Republic of Congo (former Zaire; EBOV1976-GP) and the GP of an isolate circulating in West Africa in 2014 (EBOV2014-GP).

As cells representing the natural reservoir of filoviruses we chose cell lines established from the Egyptian fruit bat (*Rousettus aegyptiacus*; RoNi/7), the hammer-headed flying fox (*Hypsignathus monstrosus*; HypNi/1.1), the straw- colored fruit bat (*Eidolon helvum*; EidNi/41) and Buettikofer's epauletted fruit bat (*Epomops buettikoferi*; EpoNi/22.1) (Table 1). Those fruit bat species are either a proven filovirus reservoir (*Rousettus aegyptiacus* for MARV) or are in the discussion as one (*Hypsignathus monstrosus* for MARV and EBOV, *Eidolon helvum* for EBOV) [14, 55]. Since it was not possible to obtain a cell line from Franquet's epauletted fruit bat (*Epomops franqueti*), another species of fruit bat linked to filoviruses (MARV and EBOV) [55], we instead used a cell line from a closely related species (*Epomops buettikoferi*). The overall geographic distribution of the four fruit bat species ranges from rather concentrated habitats at the southern coast of West Africa (*Epomops buettikoferi*) to a nearly complete coverage of the area between Sub-Saharan Africa and South Africa (Fig 1C) [56], and overlaps with sites of reported filovirus outbreaks in humans.

**Fig 2:**
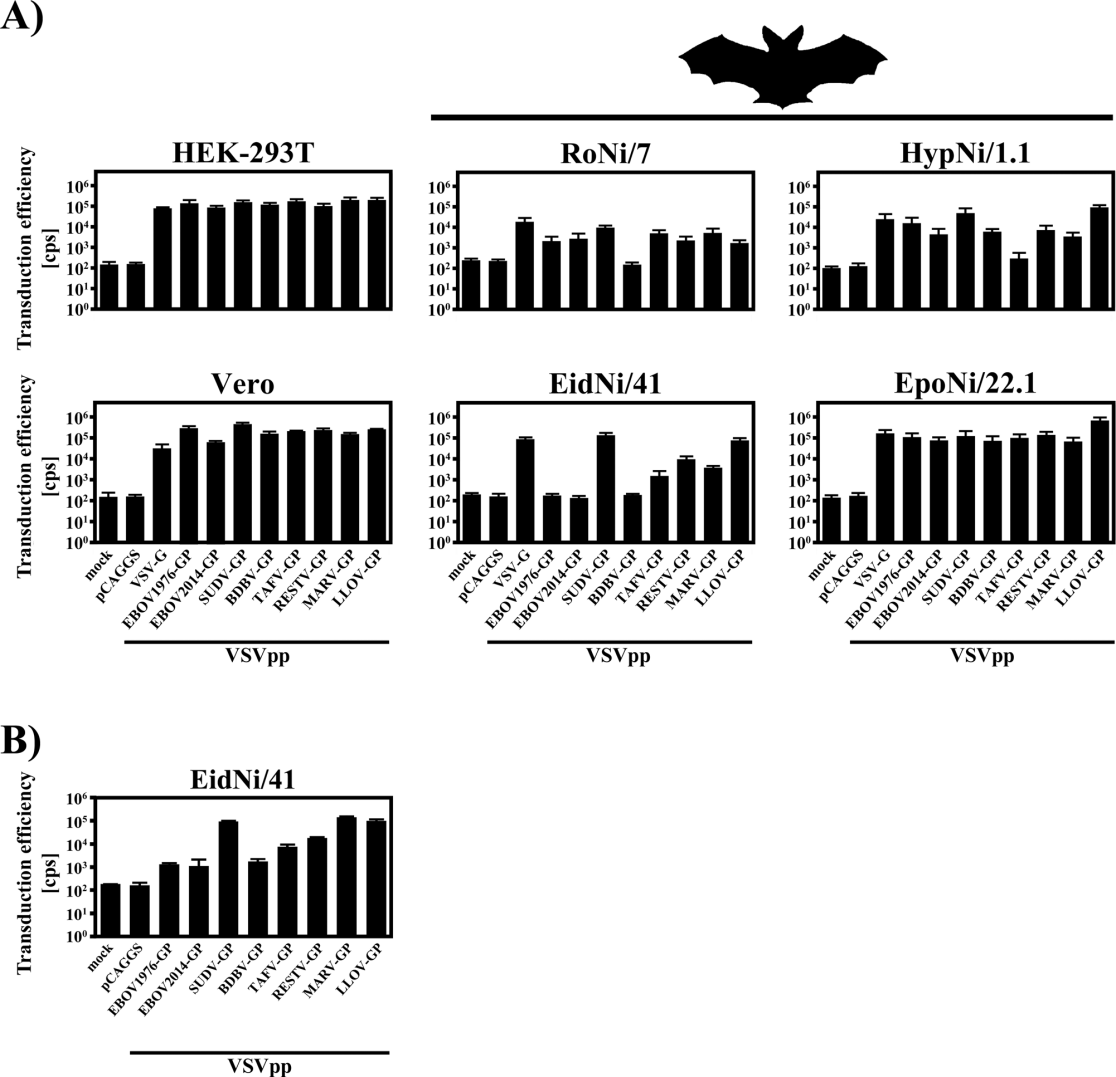
Differential transduction of fruit bat cells lines by different filovirus glycoproteins. Vesicular stomatitis virus (VSV)-based pseudotypes (VSVpp) harboring the indicated filovirus glycoproteins or the glycoprotein of VSV (VSV-G; positive control), or no glycoprotein (pCAGGS; negative control) were used to inoculate human (HEK-293T), non-human primate (Vero) and fruit bat cell lines (RoNi/7, HypNi/1.1, EidNi/41, EpoNi/22.1). Mock-treated cells that received only fresh culture medium served as additional negative controls (mock). The VSVpp decorated with the respective GPs or VSV-G were either infectivity-normalized (A) or applied undiluted (B) to the target cells. At 18 h post inoculation, the activity of virus-encoded firefly luciferase (given in counts per second; cps) as an indicator for transduction efficiency was quantified. The results of a single representative experiment carried out with quadruplicate samples is shown. Similar results were obtained in three independent experiments carried out with separate pseudotype preparations. Error bars indicate standard deviations (SD).

In order to assess transduction efficiency, we inoculated human (HEK-293T), non-human primate (Vero) and four fruit bat cell lines with VSVpp harboring filovirus GPs or VSV-G as control. First, we normalized the VSVpp for comparable transduction of HEK-293T cells and then used the particles for transduction of primate and bat cell lines (Fig 2A). For Vero cells, the transduction mediated by filovirus GPs was roughly comparable to that measured for HEK-293T cells. However, the efficiency of entry mediated by the EBOV2014-GP was notably lower (~80%) than for the EBOV1976-GP, which is in line with previous results obtained for another African green monkey-derived cell line, COS-7 [47]. EpoNi/22.1 bat cells were also comparably susceptible to transduction by all GPs, although LLOV-GP-mediated entry was slightly increased (Fig 2A). For the remaining three bat cell lines, marked differences in transduction by GPs representing different filovirus species were observed. Transduction of RoNi/7 and HypNi/1.1 cells by BDBV-and TAFV-GP bearing particles, respectively, was markedly reduced compared to transduction driven the other GPs (Fig 2A). Even more profound differences were observed for EidNi/41 cells. The GPs of SUDV and LLOV facilitated robust transduction of the cells while both EBOV-GPs tested (1976 and 2014) as well as BDBV-GP failed to mediate entry into this cell line. TAFV-, RESTV- and MARV-GP-bearing VSVpp displayed intermediate transduction efficiency. Finally, when undiluted VSVpp were used for inoculation of EidNi/41 cells, we could detect low levels of luciferase activity, indicating that also EBOV- and BDBV-GP are capable of mediating entry into cells of *Eidolon helvum*, albeit with low efficacy (Fig 2B). In sum, the GPs of filovirus species differ widely in their ability to transduce target cells of certain reservoir species.

### Furin cleavage site and the mucin-like domain are dispensable for EBOV-GP-driven entry into fruit bat cell lines

**Fig 3:**
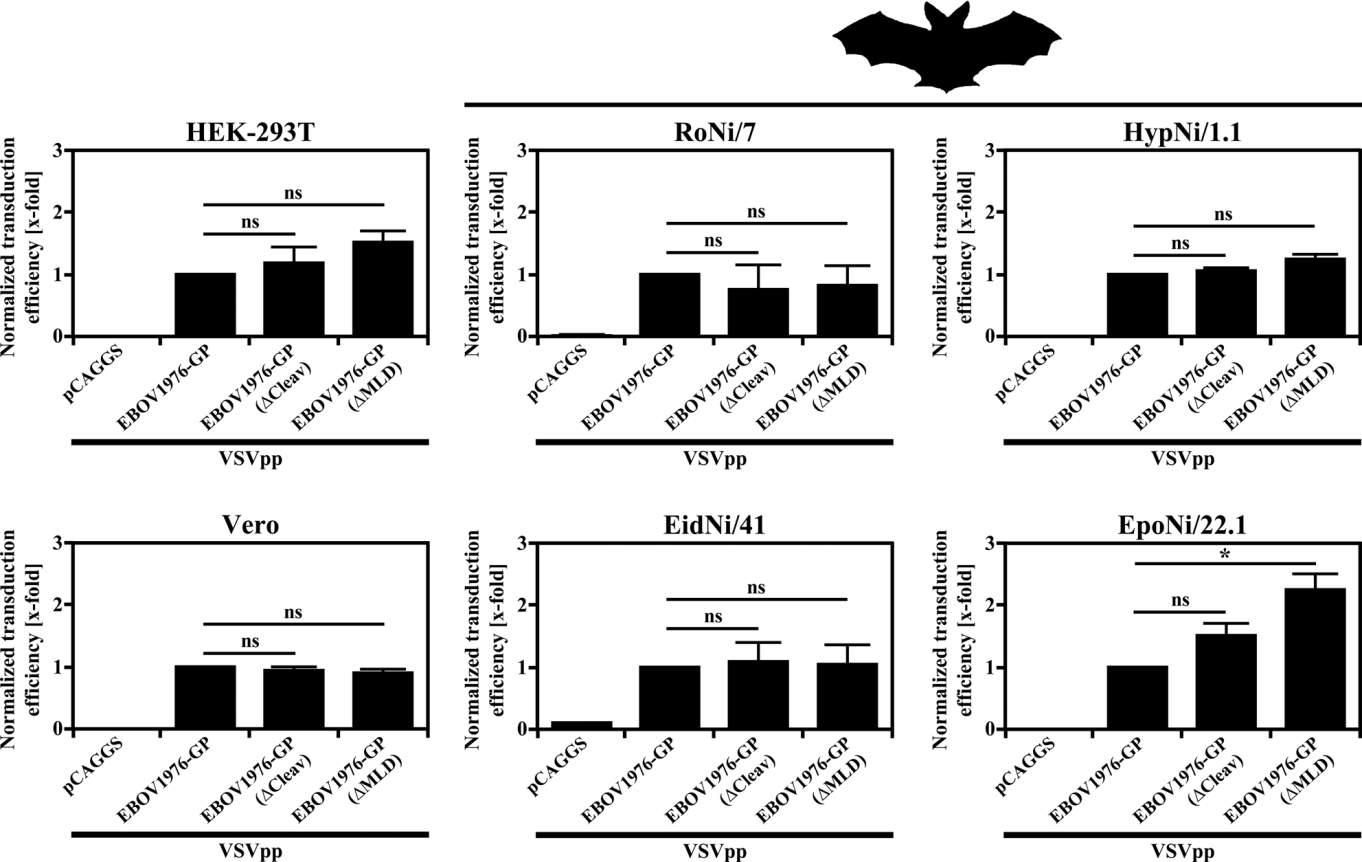
Furin cleavage and mucin-like domain are dispensable for EBOV-GP-driven transduction of fruit bat cells. Equal volumes of vesicular stomatitis virus (VSV)-based pseudotypes (VSVpp) harboring the indicated glycoproteins were inoculated onto human (HEK-293T), primate (Vero) and fruit bat cell lines (RoNi/7, HypNi/1.1, EidNi/41, EpoNi/22.1). EBOV1976-GP(∆Cleav) cannot be cleaved by furin while EBOV1976-GP(∆MLD) does not contain the mucin-like domain. VSVpp that did not harbor a viral glycoprotein at all served as negative controls (pCAGGS). At 18 h post inoculation, the activity of virus-encoded firefly luciferase in cell lysates was quantified as an indicator for transduction efficiency. Transduction mediated by the tested GPs is shown relative to transduction mediated by wt EBOV1976-GP, which was set as 1. The average of three independent experiments with separate pseudotype preparations is shown. Error bars indicate standard error of the mean. A paired student’s t-test was performed to test statistical significance (* = p < 0.05).

We next addressed whether the furin cleavage site and MLD, which are dispensable for entry into humans cells [23, 24, 57–60], might be required for entry into bat cells. For this, we employed two EBOV-GP mutants: EBOV-GP(∆Cleav), in which the furin cleavage motif was mutated (_497_RRTRR_501_ > _497_AGTAA_501_) and cleavage was abolished [24], and EBOV-GP(∆MLD), which lacks almost the entire MLD (aa residues 309–486, Fig 1B). The GP mutants facilitated entry into human, primate and fruit bat cell lines with comparable efficiency as GP wt (Fig 3). Moreover, transduction of HEK-293T and one of the four fruit bat cell lines by particles bearing the MLD-deletion mutant was slightly higher than that measured for GP wt. These findings are in keeping with data documented for human cell lines [58–60] although previous studies documented a more robust effect, possibly due to differences in the pseudotyping system. Thus, furin cleavage motif and MLV are dispensable for GP-mediated transduction of human, primate and bat cells.

### Glycoprotein-driven entry into fruit bat and human cell lines depends on the same host cell factors

We next investigated whether GP-mediated entry into fruit bat cells depends on the same host cell factors as described for human cells. To this end, we incubated human (HEK-293T) and fruit bat cell lines with inhibitors interrupting discrete steps of the entry process before pseudotypes harboring either of the filovirus GPs were added. We chose the fruit bat cell lines EpoNi/22.1 and EidNi/41 for these studies since they either showed a comparable susceptibility for transduction by all tested GPs or displayed varying susceptibility depending on which particular filovirus GP was used, respectively. For EidNi/41 cells, we only included VSVpp harboring SUDV-, TAFV-, RESTV-, MARV- and LLOV-GP, since transduction of pseudotypes decorated with EBOV1976-, EBOV2014- or BDBV-GP was only slightly above the limit of detection (Fig 1B). On the side of the host cell factors we chose inhibitors (Table 2) that targeted cellular lectins (mannan), endosomal acidification and thus indirectly cysteine protease activity (ammonium chloride, bafilomycin A1), cysteine proteases (E-64d; MDL28170), serine proteases (camostat mesylate), two-pore channels (tetrandrine) and NPC1 (U18666A, a compound that was recently shown to directly bind to NPC1 [61]). Except for the serine protease inhibitor an inhibitory effect of these agents on GP-mediated entry into mammalian cells has been previously described [32, 35, 37, 62–65].

**Fig 4:**
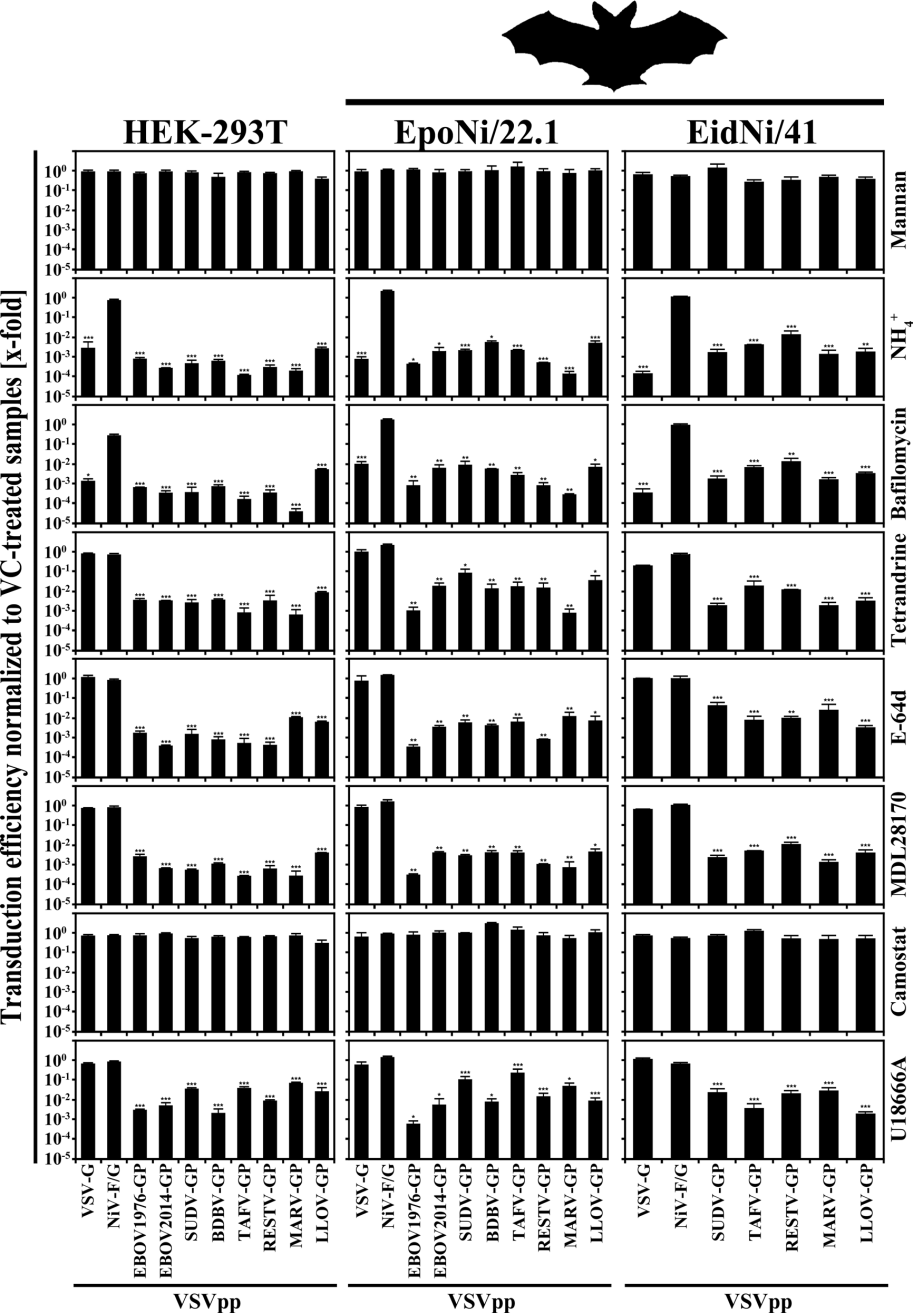
Glycoprotein-mediated entry into human and fruit bat cells relies on the same host cell factors. Equal volumes of vesicular stomatitis virus (VSV)-based pseudotypes (VSVpp) harboring the indicated glycoproteins were used to inoculate human (HEK-293T) and fruit bat (EpoNi/22.1, EidNi/41) cell lines pre-incubated with the indicated inhibitors for 3 h. Cells treated with solvent (water or dimethyl sulfoxide) alone served as controls (vehicle control, VC). At 18 h post inoculation, the activity of virus-encoded firefly luciferase as an indicator for transduction efficiency was quantified and normalized against the values of the respective VC (x-fold changes). The results of a representative experiment carried out with quadruplicate samples are shown and were confirmed in an independent experiment, conducted with a separate pseudotype batch. The following inhibitor concentrations were used: Mannan (final concentration: 25 μg/ml), ammonium chloride (NH_4_^+^; 50 mM), bafilomycin A1 (50 nM), tetrandrine (2 μM), E-64d (50 μM), MDL28170 (50 μM), camostat mesylate (100 μM), U18666A (20 μM). Error bars indicate SD. An unpaired student’s t-test was used to test statistical significance (* = p < 0.05, ** = p < 0.01, *** = p < 0.001).

In comparison to cells treated only with diluent (vehicle control, VC), we found that ammonium chloride, bafilomycin A1, E-64d, MDL28170, tetrandrine and U18666A inhibited entry driven by all filovirus GPs tested, while mannan and camostat mesylate had no appreciable inhibitory effect (Fig 4). Inhibition of GP-mediated entry into bat cells by ammonium chloride, bafilomycin A1, tetrandrine E-64d and MDL28170 seemed to be slightly less efficient relative to human cells but no clear differences were observed. In comparison, transduction mediated by VSV-G was reduced only by agents interfering with endosomal acidification (Fig 4), in accordance with published data [70]. Finally, entry of VSVpp harboring NiV-F/G, which were used as an additional control, was not inhibited by any of the compounds tested (Fig 4). NiV-F needs to undergo proteolytic cleavage by the cysteine proteases cathepsin B or L. However, this step does not take place during the entry process but rather during transport of F protein to the plasma membrane and was not targeted under the experimental conditions chosen here [71]. Collectively, our observations indicate that filovirus GPs use the same cellular factors for entry into human, NHP and bat cell lines.

## Discussion

Filoviruses, in particular ebola- and marburgviruses, pose a severe threat to humans and wildlife [5], not only in Africa but, as a result of increased international travel and trade, also globally. This is highlighted by the currently ongoing EVD epidemic in West Africa, where for the first time a filovirus outbreak spread from remote to densely populated areas. Bats constitute a natural reservoir for filoviruses [3, 12, 15, 55, 72] but the interaction of filoviruses with bat cells remains largely uncharacterized. The present study addressed the question whether filovirus entry into cells from the natural reservoir, fruit bats, relies on the same molecular mechanisms as entry into human and NHP cells. We found that GPs of all filovirus species are able to mediate entry into bat cells but differ in their efficacy. Furthermore, GP processing by the host protease furin and presence of the MLD were dispensable for efficient entry into bat cells, while blockade of host factors known to be important for filovirus entry into human cells (including cysteine proteases, two pore channels and NPC1) also reduced entry into bat cells. Taken together, our results indicate that filovirus entry into human and fruit bat cells relies on the same host cell factors, although the capacity to engage those factors seems to differ among the GPs of the filovirus species.

We employed rhabdoviral pseudotypes harboring GPs representing all filovirus species (EBOV, SUDV, BDBV, TAFV, RESTV, MARV, and LLOV) to study GP-mediated entry. Our results point towards pronounced differences in susceptibility of fruit bat cell lines to entry driven by filovirus GPs: Cells from the kidney of a Buettikofer's epauletted fruit bat (*Epomops buettikoferi*, EpoNi/22.1) were comparably susceptible to transduction mediated by all filovirus GPs tested. In contrast, cells from the Hammer-headed fruit bat (*Hypsignathus monstrosus*, HypNi/1.1) and the Egyptian fruit bat (*Rousettus aegyptiacus*, RoNi/7), displayed a reduced susceptibility for TAFV-GP- and BDBV-GP-driven pseudotype entry, respectively. The results obtained for transduction of RoNi/7, HypNi/1.1 and EpoNi/22.1 by EBOV-, SUDV-, RESTV- and MARV-GP are in keeping with and expand previous studies [39, 42, 47]. More strikingly, we observed remarkable differences when GP-mediated transduction of a cell line was investigated that has been established from the kidney of a Straw-colored fruit bat (*Eidolon helvum*, EidNi/41). VSVpp decorated with either of the two EBOV-GPs or BDBV-GP failed to appreciably transduce these cells, while all other GPs mediated moderate (TAFV-, RESTV-, MARV-GP) to robust (SUDV-, LLOV-GP) transduction. During the revision of this manuscript Ng and colleagues reported inefficient EBOV-GP-driven entry into cells from the straw-colored fruit bat as compared to cells from other bats [73]. The inefficient entry was linked to a single amino acid polymorphism within the NPC1 molecule of this particular bat species [73]. These findings and the results of the present study indicate that filovirus entry into reservoir cells can be restricted at the stage of NPC1 usage. Moreover, they suggest that GPs representing different filovirus species might engage bat NPC1 orthologues slightly differently.

LLOV-GP mediated highly efficient entry into three out of four fruit bat cell lines (HypNi/1.1, EidNi/41, EpoNi/22.1), in line with a previous study reporting superior transduction efficiency by LLOV-GP (relative to EBOV-, RESTV- and MARV-GP) when bat cell lines were examined [50]. In addition, we were able to confirm previous data that indicated reduced transduction of NHP cells by EBOV2014-GP compared to EBOV1976-GP [47]. This observation is in line with the finding that cynomolgus macaques experimentally infected with the Makona strain of EBOV (i.e. EBOV2014) showed delayed disease progression compared to animals inoculated with EBOV1976 [74]. Collectively, these findings highlight that filovirus GPs can differ markedly in their capacity to mediate entry into cell lines of various species. This finding reflects the diverse nature of the different filovirus species that, despite their taxonomical relatedness, may engage certain host cell-encoded entry factors with different efficiencies.

A MLD and a furin-cleavage site are present in all filovirus GPs. Nevertheless, these elements are dispensable for entry into human cells lines and, in case of the furin cleavage site, for viral spread and pathogenesis in the host [23, 24, 57–60]. A scenario accounting for these discrepant findings could be that these elements are required for entry into reservoir but not NHP and human cells. The present study shows that this is not the case, since neither inactivation of the cleavage site nor deletion of the MLD appreciably altered transduction of the fruit bat cell lines investigated. One could speculate that the MLD is required for efficient spread in the host, potentially by modulating immune recognition of GP, and is thus present in all filovirus GPs. Why the GP motif for processing by furin or other proprotein convertases is conserved requires further investigation.

We employed inhibitors of cellular factors or processes known to be required for filovirus GP-driven entry into human cells to investigate whether filoviruses hijack the same factors/process to enter bat cells. We observed a profound decrease in GP-mediated transduction when endosomal acidification (inhibited by ammonium chloride or bafilomycin A1), calcium-dependent two-pore channels (inhibited by tetrandrine), cysteine proteases such as cathepsins and calpain (inhibited by E-64d and MDL28170) were blocked. Similarly, U18666A treatment, which induces an NPC1 knock-out phenotype, reduced entry into all cell lines tested, in line with the published finding that LLOV-GP requires NPC1 for entry into bat cell lines [38]. In general, no appreciable differences in the effectiveness of the inhibitors between human (HEK-293T) and fruit bat (EpoNi/22.1 and EidNi/41) cells were observed. A slightly less efficient block of GP-mediated entry into bat cells by agents interfering with endosomal acidification as well as the activity of TPCs and cysteine proteases might result from species specific differences in the drug targets. Additionally, it cannot be ruled out that the usage of more specific cysteine protease inhibitors would reveal subtle differences in protease choice of EBOV in human and bat cells. Finally, there were no obvious differences between bat cells that either showed a comparable (EpoNi/22.1) or differential (EidNi/41) susceptibility for transduction by the seven GPs. These observations suggest that all filovirus GPs depend on the same host cell factors for entry into human and fruit bat cell lines.

The mannose-polymer mannan and the serine protease inhibitor camostat mesylate did not inhibit filovirus GP-driven entry into any of the cell lines tested. Mannan was previously shown to block augmentation of GP-driven entry by DC-SIGN and other mannose-binding lectins [62, 75], which are endogenously expressed on a subset of primary filovirus target cells. The most straightforward explanation for the inability of mannan to block entry in the present study is thus that none of the cell lines tested expressed mannose-specific lectins able to augment filovirus entry. Camostat mesylate inhibits activation of influenza A virus and coronavirus glycoproteins by the type II transmembrane serine protease TMPRSS2 [76–79]. Filovirus GPs are activated by the pH dependent endosomal cysteine proteases cathepsin B and L, at least in several target cell lines [34, 35], and that lack of inhibition of GP-driven entry by camostat mesylate was thus not unexpected.

In sum, the present study suggests that filoviruses do not need to adapt their entry mechanism to successfully jump between bats, NHPs and humans. However, differences in the efficiency with which filovirus species infect certain reservoir hosts might exist and might be due to polymorphisms in NPC1 [73] and/or additional host cell factors.
